# Ethylene signaling modulates contents of catechin and ability of antioxidant in *Camellia sinensis*

**DOI:** 10.1186/s40529-018-0226-x

**Published:** 2018-04-03

**Authors:** Shun-Wun Ke, Guan-Heng Chen, Chung-Tse Chen, Jason T. C. Tzen, Chin-Ying Yang

**Affiliations:** 1 Department of Agronomy, National Chung Hsing University, Taichung, 40227 Taiwan; 2 Graduate Institute of Biotechnology, National Chung Hsing University, Taichung, 40227 Taiwan

**Keywords:** Ethylene, Antioxidant, Catechins, Polyphenol, Tea plant

## Abstract

**Background:**

Tea is one of the most popular beverages in the world. There are many secondary metabolites can be found in tea such as anthocyanins, proanthocyanidins, flavonols and catechins. These secondary metabolites in plants are proved to act protective components for human health effect. Plant hormone ethylene is considered to have an important role in regulation of plant development and signal transduction. This study evaluated the effect of ethylene signaling regulation in phenolic compounds in tea plants. The ethylene precursor 1-aminocyclopropane-1-carboxylic acid (ACC) enhanced contents of total catechin in treated oolong tea seedlings.

**Results:**

The degree of epigallocatechin and epicatechin galloylation was increased after ACC treatment in oolong tea seedlings by high performance liquid chromatography determination. The contents of anthocyanins, flavonoids, and total polyphenol were higher after ACC treatment in comparison with control. Antioxidant enzyme such as catalase, superoxide dismutase, and total peroxidase decreased their antioxidant activities after ACC treatment, yet the activity of ascorbate peroxidase is increased. The ability of oxygen radical absorption and 2,2-diphenyl-1-picrylhydrazyl was used to evaluate the antioxidant activity, which was enhanced by ACC treatment.

**Conclusions:**

Taken together the results of this study demonstrate that the ethylene signaling is involved in modulation of secondary metabolites accumulation and antioxidant ability that to enhance the benefit of human health in tea products.

## Background

Tea (*Camellia sinensis* L.) is an evergreen shrub and prevalent commercial crop grown in more than 50 countries. Tea plants are rich in secondary metabolites, which have potential health benefits for humans (Zhou et al. 2016). Secondary metabolic products in tea include flavonoids, polyphenols, theanine, and alkaloids. Flavonoids are a large group of phenolic secondary metabolites including anthocyanins, proanthocyanidins, phenolic acids, flavonols and catechins (Abeynayake et al. 2012; Sun et al. 2016). The predominant flavonoids in tea are catechins (flavan-3-ols), which are generally classified into the following groups such as epicatechin (EC), epicatechin-gallate (ECG), epigallocatechin (EGC) and epigallocatechin-gallate (EGCG) (Perva-Uzunalic et al. 2006).

The production of secondary metabolites in higher plants is often affected by hormone regulation. Ethylene is a plant hormone that affects the plants growth and development and is sometimes required for processes including ripening, senescence, and abscission (Schaller 2012). In tea plants, floral buds are more sensitive to ethylene than are leaves (Woolf et al. 1999). Ethylene is critical for signal transduction and its effect on the accumulation of secondary metabolites. Ethylene regulates flavonol biosynthesis through the MYB12 transcription factor, which affects the expression of chalcone synthase, chalcone isomerase, and flavonol synthase (Lewis et al. 2011). Ethylene signaling is involved in the defense resistance of plants by enhancing polyphenol oxidase activity during stress (Bosch et al. 2014; Thipyapong and Steffens 1997). Studies on rice coleoptiles have shown that ethylene induces the accumulation of polyamine (Lee and Chu 1992).

Previous studies have shown that ethylene signaling contributes to the accumulation of secondary metabolites to facilitate defense resistance in plants. However, little is known about ethylene signaling regulation in the phenolic compounds in tea plants. 1-aminocyclopropane-1-carboxylic acid (ACC) is known to be a direct precursor of ethylene and is transported throughout the plant over short and long distances (Boller et al. 1979), it is believed that the responses observed in this study is caused by ethylene. Our results demonstrated that after treatment with the ethylene precursor ACC, the total catechin content in oolong tea seedlings increased. High-performance liquid chromatography (HPLC) indicated that the degrees of EGC and EC galloylation were increased by ACC treatment in seedlings of oolong tea. The accumulation of phenolic compounds was enhanced after ACC treatment such as anthocyanins, flavonoids, and total polyphenol. The activities of catalase (CAT), superoxide dismutase (SOD), and total peroxidase (POX) decreased and ascorbate peroxidase (APX) increased after ACC treatment by antioxidant enzyme activity assay. Ability of antioxidant was enhanced after ACC treatment via evaluated according to the oxygen radical absorption capacity (ORAC), and 2,2-diphenyl-1-picrylhydrazyl (DPPH). Our results demonstrate that the contents of secondary metabolites and capacity of antioxidant can be increased through modulating ethylene signaling pathways in tea.

## Methods

### Plant materials and treatments

Tea [*Camellia sinensis* (L.) Kuntze] cultivar Chin-Shin Oolong was used in the experiments in this study. All seedlings of the tea plant were grown in the same area in Nantou, Central Taiwan, over a 2-year period. The tea plant seedlings were approximately 50 cm high. For ACC treatment, 2-year-old tea seedlings were irrigated with 20 mL of 100 µM ACC per day for 5 days; the control (CK) samples were irrigated with 20 mL of water instead. Fresh samples each with one tip and two leaves were collected from tea seedlings, frozen immediately in liquid nitrogen, and stored at − 80 °C until analysis.

### Preparation of extracts from young tea seedling leaves

The samples were weighed, and lyophilized samples were homogenized to powder by using a disposable pestle. The extraction of tea was prepared with 1.0 mL of 75% ethanol and shaking the mixture vigorously with an Intelli-Mixer (MyLabTM RM-2 M) for 2 h at room temperature. The mixture extraction of tea was filtered by 0.45 µm polyvinylidene difluoride membrane filter (Pall Corporation, Glen Cove, NY, USA) before HPLC analysis.

### HPLC analysis

Tea extracts were analyzed on a liquid chromatography system coupled with a Model 600E photodiode array detector (Waters Corporation, Milford, MA, USA). The chromatographic separation was performed on a Mightysil RP-18 GP column (250 × 4.6 mm i.d., 5 µm; Kanto Chemical Co., Tokyo, Japan). The mobile phase consisted of water containing 0.5% acetic acid (solvent A) and acetonitrile (solvent B).

The samples were eluted using the following gradient program, 0 min, 95% A and 5% B; 100 min, 75% A and 25% B. The column was kept at room temperature, the volume of each injection was 10 µL, and the flow rate was 1 mL/min. The PDA detector was set at 280 nm. The four major catechins (EC, EGC, ECG, and EGCG) shown in the HPLC profiles were identified as described in a previous study (Chen et al. 2014).

### Determination of anthocyanins, flavonoids, and total phenol

The tea seedling samples (0.1 g) were excised and immediately used for anthocyanin, flavonoid, and total phenol content assays. For the anthocyanin and flavonoid content assays, the sample extracts in 2 mL of potassium phosphate buffer (100 mM, pH 7.8) were ground into powder with liquid nitrogen. The mixture of tea extraction was centrifuged at 16,000*g* and 4 °C for 30 min, and the resultant supernatant was measured at 600 and 320 nm by using a spectrophotometer (Metertec SP8001). One absorbance unit was defined as the quantity of anthocyanins and flavonoids that exhibits an absorbance of 1 at 600 and 320 nm. The total phenolic content assay was performed using a modified form of the procedure described by Singleton and Rossi (1965). The sample extracts in 1.0 mL of 75% ethanol were ground into powder with liquid nitrogen. The homogenate was centrifuged at 10,000*g* and 4 °C for 10 min, and 50 μL of the resultant supernatant was diluted with distilled water and added to 250 μL of the Folin–Ciocalteu reagent (0.2 mol/L). The reaction mixture reacted for 5 min before 50 μL of 20% Na_2_CO_3_ was added. The samples were then incubated at room temperature in darkness for 60 min, and the absorbance was measured at 760 nm. A calibration curve was obtained using 0–4000 mg of gallic acid per milliliter and used to calculate the total phenolic content of the tea seedling leaves.

### Determination of antioxidant enzyme activity

The fresh leaves (0.2 g) of tea seedling were used immediately for enzyme extraction after excised, and subsequently homogenized with liquid nitrogen. Sodium phosphate buffer (50 mM; pH 6.8) was used as extraction buffer. The mixture of tea leaves extraction was then centrifuged at 15,000*g* for 30 min, and the resultant supernatant was used in the following enzyme activity assays. CAT activity was analyzed as described in previous studies (Chao et al. 2012; Kato and Shimizu 1985). Decreasing in absorbance at 240 nm was observed using a spectrophotometer (Metertec SP8001) which implied the decrease of H_2_O_2_. Activity of CAT was calculated on the basis of the extinction coefficient (40 mM^−1^ cm^−1^ at 240 nm) of H_2_O_2_. One unit of CAT was defined as the enzyme amount that degraded 1 μmol of H_2_O_2_ per minute. SOD activity assay was conducted based on Chao et al. (2012). The reaction buffer was Triethanolamine–diethanolamine buffer (100 mM; pH 7.4) containing ethylenediaminetetraacetic acid/MnCl_2_ (100 mM/50 mM, pH 7.4), 7.5 mM β-nicotinamide adenine dinucleotide (β-NADH), and 10 mM 2-mercaptoethanol and mixed with enzyme extract. The enzyme reaction was initiated by the addition of β-NADH, and detected the absorbance at 340 nm for 10 min. One unit of SOD was defined as the enzyme amount that inhibited the rate of β-NADH oxidation by 50%. For APX activity assay, the decrease in the ascorbic acid (AsA) concentration was determined according to the decline in absorbance at 290 nm, and the activity was calculated based on the extinction coefficient (2.8 mM^−1^ cm^−1^ at 290 nm) for AsA (Chao et al. 2012; Nakano and Asada 1981). Both soluble and ionically bound peroxidase (Total POX) activity assay was modified from previous studies (Lin and Kao 1999; MacAdam et al. 1992; Wu and Yang 2016). Samples were extracted by homogenized in liquid nitrogen with 50 mM potassium phosphate buffer (pH 5.8) containing 0.8 M KCl buffer. The 50 mM potassium phosphate buffer (pH 5.8), 21.6 mM guaiacol, and 39 mM H_2_O_2_ were added into enzyme extract for reaction. The enzyme reaction was initiated by adding H_2_O_2_, the absorbance was measured at 470 nm for 3 min. Total POX activity was calculated on the basis of the extinction coefficient of 26.6 mM^−1^ cm^−1^ at 470 nm for tetraguaiacol. One unit of POX was defined as the enzyme amount that caused the formation of 1 μmol of tetraguaiacol per minute.

### Determination of DPPH radical scavenging activity

Potential antioxidant activity was determined using DPPH (2,2-diphenyl-1-picrylhydrazyl) according to Tadolini et al. (2000) with some modifications. The sample (0.1 g) extract was added to 1.0 mL of 75% ethanol. The mixture was shaken for 120 min through vortexing and left to centrifuge at 10,000*g* and room temperature in darkness for 10 min. The absorbance for the sample was measured using a SpectraMax M2 spectrophotometer at 521 nm against an ethanol blank. A control sample (ΔA_DPPH_) was extracted after adding 0.19 mM DPPH solution to 0.2 mL of the respective extraction solvent. Every sample was extracted in triplicate, and the results were calculated based on four biologically independent experiments. The percentage of DPPH free radicals scavenged in the sample was calculated using the following equation:

$${\text{DPPH free radical scavenging ratio }}\left( \% \right) \, = \, \left[ {{{\left( {\Delta {\text{A}}_{\text{DPPH}} - \Delta {\text{A}}_{{{\text{sample}} + {\text{DPPH}}}} } \right)} \mathord{\left/ {\vphantom {{\left( {\Delta {\text{A}}_{\text{DPPH}} - \Delta {\text{A}}_{{{\text{sample}} + {\text{DPPH}}}} } \right)} {\left( {\Delta {\text{A}}_{\text{DPPH}} } \right)}}} \right. \kern-0pt} {\left( {\Delta {\text{A}}_{\text{DPPH}} } \right)}}} \right] \, \times 100$$

### Oxygen radical absorbance capacity (ORAC) measurement

The ORAC assay was by using a modified form of the method described by Ou et al. (2001). The ORAC assay was measured the capacity of antioxidative compounds in test materials to inhibit the decrease in fluorescence induced by the peroxyl radical 2,2′-azobis (2-amidinopropane) dihydrochloride (AAPH). The samples (0.1 g) were homogenized with liquid nitrogen and added to 1 mL of double distilled H_2_O for incubation 10 min at 100 °C. The 20 μL extract was added to 120 μL 120 nM disodium fluorescein solution (72 nM, final concentration) and preincubated for 15 min at 37 °C. The mixture was rapidly added to 60 μL of 40 mM AAPH in the well of the microplate and the microplate was immediately placed in the reader, and the fluorescence was recorded at an 485 nm (excitation wavelength) and 535 nm (emission wavelength) every 1 min for 60 min. The blank (FL + AAPH) with phosphate buffer instead of the antioxidant solution and Trolox was used as the standard. All the reaction mixtures were detected in duplicate, and at least three independent assays were performed for each sample. Fluorescence was measured and recorded every 1 min (*f*0, *f*1, *f*2, *f*3,…, *f*60) until the fluorescence began to decline. The curves of antioxidant were initially normalized to the blank curve corresponding to the same assay by the factor fluorescence_blank,t= 0_/fluorescence_sample,t=0_. Based on the normalized curves, the area under the fluorescence decay curve (AUC) was calculated as follows:

$${\text{AUC = 1 + }}\sum\limits_{i = 1}^{i = 60} {{{fi} \mathord{\left/ {\vphantom {{fi} {f0}}} \right. \kern-0pt} {f0}}}$$

$${\text{ORAC}}_{\text{FL}} = \frac{{{\text{AUC}}_{\text{sample}} - {\text{AUC}}_{\text{blank}} }}{{{\text{AUC}}_{\text{trolox}} - {\text{AUC}}_{\text{blank}} }} \times {\text{Trolox}}\;{\text{molarity}} \times {\text{sample}}\;{\text{dilution}} .$$

Each sample was calculated by subtracting the AUC corresponding to the blank. Equations for regression between the net AUC and antioxidant concentration were calculated for all samples. The concentrations are expressed as micrograms of Trolox equivalents per 100 mg of fresh weight.

## Results

### Content and degree of catechin galloylation were higher in response to ethylene signaling

**Fig. 1:**
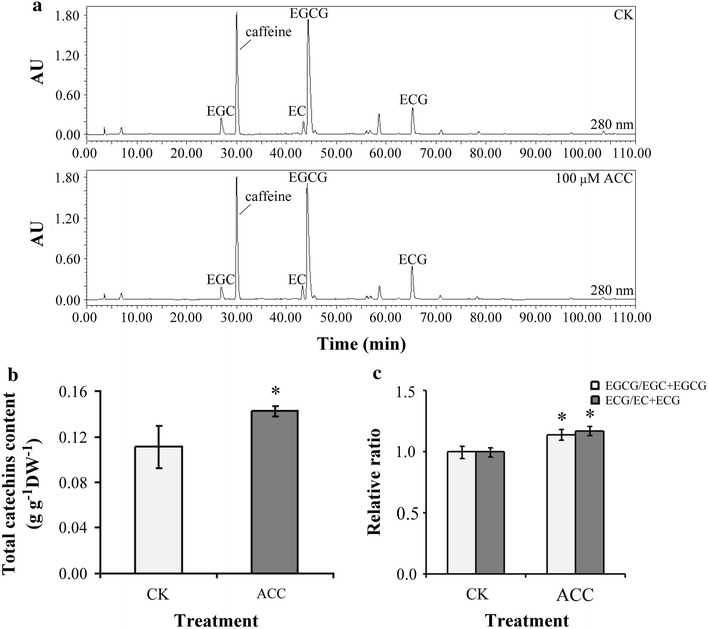
Effects of ethylene signaling on the content and degree of catechin galloylation in tea seedlings. **a** Caffeine and the four major catechins, EC, EGC, ECG, and EGCG, were detected. **b** Total catechin content. **c** The relative ratio was calculated as follows: ECG/(EC + ECG) and EGCG/(EGC + EGCG). Data points are mean ± SD of five biologically independent experiments. Asterisks indicate significant differences from the control sample (P < 0.05; Student’s *t* test)

The level of catechin galloylation was determined through HPLC after 5 days of treatment with the ethylene precursor ACC, and the content of total catechin and values of ECG/(EC + ECG) and EGCG/(EGC + EGCG) were calculated. The HPLC profiles revealed the corresponding peaks to caffeine and the four major catechins including EC, ECG, EGC, and EGCG (Fig. 1a). The total catechin content was higher in the tea seedlings treated with ACC than in the control sample (Fig. 1b). The degrees of EGC and EC galloylation were higher after ACC treatment in comparison with the control sample (Fig. 1c).

### Content of anthocyanins, flavonoids, and total polyphenol was enhanced after the ACC treatment

**Fig. 2:**
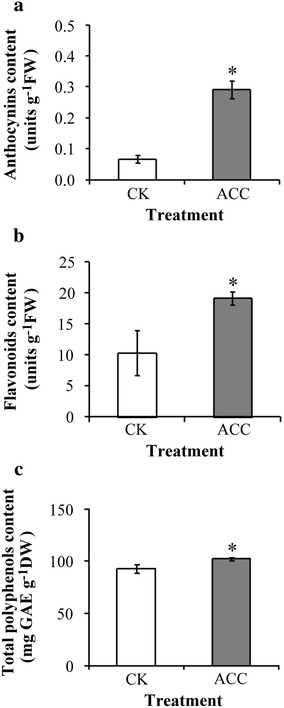
Effects of ethylene signaling on the content of secondary metabolites in tea seedlings. The content of **a** anthocyanins, **b** flavonoids, and **c** total phenol was determined. Data points are mean ± SD of three biologically independent experiments. Asterisks indicate significant differences from the control sample (P < 0.05; Student’s *t* test)

To investigate the effects of ethylene signaling in the secondary metabolic pathway, the contents of anthocyanins, flavonoids, and total polyphenol were determined after ACC treatment in tea seedlings. The contents of anthocyanins (Fig. 2a), flavonoids (Fig. 2b), and total polyphenols (Fig. 2c) were higher in the tea seedlings that underwent ACC treatment than in the control sample. These findings show that ethylene signaling was involved in the modulation of the metabolic pathway in the oolong tea seedlings.

### Effects of ethylene signaling on antioxidant enzyme activity

**Fig. 3:**
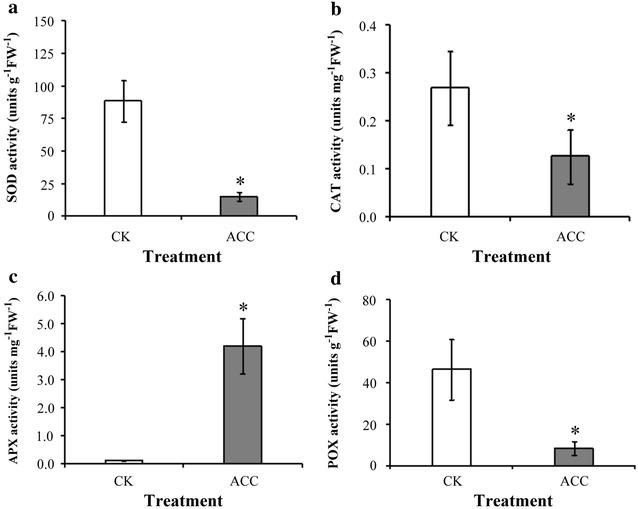
CAT, APX, SOD, and POX activity in tea seedlings on modulation of ethylene signaling. Enzyme activity was detected from the detached tea seedling samples with one tip and two leaves after treatment with the control (CK) or 100 μM ACC for 5 days.CAT, APX, SOD, and total POX activity was determined. Data points are mean ± SD of five biologically independent experiments. Asterisks indicate significant differences from the control sample (P < 0.05; Student’s *t* test)

To evaluate the cellular free radical scavenging capacity of the antioxidant enzymes in oolong tea seedlings after ACC treatment, the activity of antioxidant enzymes such as SOD, CAT, APX, and total POX was evaluated. Our results show that the activity of SOD (Fig. 3a), CAT (Fig. 3b), and POX (Fig. 3d) decreased under ACC treatment for 5 days. By contrast, APX activity significantly increased under ACC treatment for 5 days (Fig. 3c).

### Antioxidant capacity was increased on modulation of ethylene signaling

**Fig. 4:**
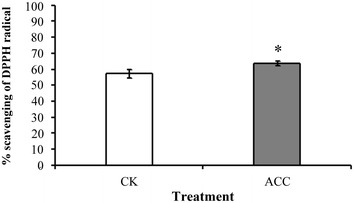
DPPH radical scavenging activity in tea seedlings on modulation of ethylene signaling. The scavenging ratios of DPPH radicals were determined from the detached tea seedling samples after treatment with the control (CK) or 100 μM ACC for 5 days. Data points are mean ± SD of three biologically independent experiments. Asterisks indicate significant differences from the control sample (P < 0.05; Student’s *t* test)

**Fig. 5:**
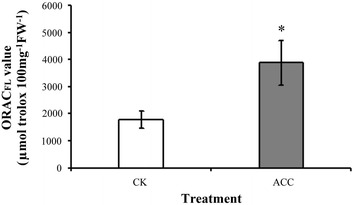
ORAC in tea seedlings on modulation of ethylene signaling. ORAC-FL values were determined from the detached tea seedling samples after treatment with the control (CK) or 100 μM ACC for 5 days. Data points are mean ± SD of three biologically independent experiments. Asterisks indicate significant differences from the control sample (P < 0.05; Student’s *t* test)

To evaluate the effects of ethylene signaling on the antioxidant capacity of tea seedlings, assays of DPPH and ORAC were conducted. After ACC treatment for 5 days, 64% of the DPPH radicals were scavenged, whereas only 57% were scavenged in the control sample (Fig. 4). The ORAC-FL assay indicated that the ORAC was enhanced by ACC treatment (Fig. 5).

## Discussion

### Ethylene signaling involved in the flavonoid synthesis pathway in tea seedlings

Ethylene is a vital phytohormone in plants. Some previous studies have demonstrated that ethylene signaling is involved in metabolic pathways; for example, ethylene treatment increased total polyphenol and anthocyanin after grape harvesting (Bellincontro et al. 2006). Studies have indicated that exogenous application of α-aminoisobutyric acid as an analogue of ACC maintained higher catechin content in longan fruit pericarp during storage (Wang et al. 2015). Catechins are the most vital secondary metabolites in tea plants which already demonstrated galloylated catechins presented stronger positive health benefit potential than that of nongalloylated catechins (Chen et al. 2014). The results of this study show increases in not only the total catechin content but also the level of EGC and EC galloylation after ACC treatment in seedlings of oolong tea (Fig. 1). Moreover, the content of anthocyanins, flavonoids, and total polyphenol was enhanced by ACC treatment in oolong tea seedlings (Fig. 2). Our results indicate that ethylene signaling was involved in the flavonoid biosynthesis pathway and modulated the catechin galloylation level in oolong tea seedlings.

### Role of ethylene signaling in antioxidant enzyme activity and antioxidant capacity in oolong tea seedlings

Reactive oxygen species (ROSs) are produced in chloroplasts, mitochondria, and peroxisomes as a typical product of plant cellular metabolism. Several studies have reported that plants exhibited excessive ROS production when they were subjected to biotic or abiotic stress, and the consequent oxidant–antioxidant imbalance activated factors responsible for cell injury (Liu et al. 2013; Wang et al. 2012; Wu and Yang 2016). Antioxidants are crucial because their main responsibility is the scavenging of ROSs, which is indispensable in protecting cell constituents from damage. Studies on phenolic compounds have shown that they are involved in plant antioxidant defense systems (Posmyk et al. 2009). Ethylene is known involved in the abscission of vegetative and reproductive organs and senescence under stress conditions. Ethylene reduced chilling damage during fruit storage and increased the activity of APX enzymes (Lafuente et al. 2004). Our data showed that using the ethylene precursor ACC to treat tea seedlings promoted the induction of phenolic compounds (Fig. 2). Therefore, ethylene signaling presumably affects the tea plant antioxidant system balance. The antioxidant enzyme activity assay revealed that the SOD, CAT, and POX activity in tea seedlings (Fig. 3a, b, d, respectively) was decreased after ACC treatment. By contrast, the APX activity was significantly increased after ACC treatment (Fig. 3c). These findings suggest that ethylene signaling promoted higher phenolic compound accumulation, which has implications for antioxidant enzyme activity in tea seedlings. The antioxidant capacity was determined by evaluating ORAC and DPPH free radical scavenging (Figs. 4 and 5). Our results indicate that higher antioxidant capacity in oolong tea seedlings can be achieved through ethylene signaling.

## Conclusions

According to our results suggest that the ethylene signaling involved in the secondary metabolic pathway induced the accumulation of phenolic compounds and catechin galloylation and reduced antioxidant enzyme activity in the tea seedlings. The antioxidant capacity was positively correlated with phenolic compound accumulation during ethylene signaling. Whether or not the ACC signal is possible involved to regulate plant secondary metabolic pathway requires further research.

## Abbreviations

ACC: 1-aminocyclopropane-1-carboxylic acid
EC: epicatechin
ECG: epicatechin-gallate
EGC: epigallocatechin
EGCG: epigallocatechin-gallate
HPLC: high-performance liquid chromatography
CAT: catalase
SOD: superoxide dismutase
POX: total peroxidase
APX: ascorbate peroxidase
ORAC: oxygen radical absorption capacity
DPPH: 2,2-diphenyl-1-picrylhydrazyl
ROS: reactive oxygen species
